# Supplementary material for: Maternal expression and breast milk transfer of an mRNA- encoded monoclonal antibody in a murine model of cholera
Source: PLoS Negl Trop Dis. 2026 Feb 9;20(2):e0013518. doi: 10.1371/journal.pntd.0013518 (PMC12919922; doi:10.1371/journal.pntd.0013518)
Supplement: Attachment — Submitted filename: R2R_PNTD-D-25-01553_R1.docx [file pntd.0013518.s002.docx]

**Response to Reviewers (PNTD-D-25-01553_R1)**

**Additional Editor Comments (if provided):** Each reviewer found the work to be novel and significant. Nevertheless, each also asked for revisions. I hope that you find the reviews helpful in drafting a revised manuscript.

**Reviewers' Comments by sections:**

**METHODS**

**Reviewer #1:**

**1.1 The objectives are novel and well-motivated, but the central hypothesis (that maternal mRNA-LNP administration can confer mucosal protection to pups) is not explicitly framed as testable.** Response: We have clarified the study’s central hypothesis in the **Introduction** to explicitly link objectives to measurable outcomes. **Line 90 now reads:** “Therefore in this study, we tested whether maternal administration of ZAC-3 mRNA-LNPs could induce functional antibody expression in serum and milk, enable transfer to offspring, and reduce *V. cholerae* colonization, thereby demonstrating the potential of this mRNA platform to deliver protective antibodies via breast milk.”

**1.2 The design demonstrates maternal-to-infant antibody transfer but does not directly test protective efficacy against *V. cholerae*, leaving a gap between objectives and outcomes.** Response: We now clarify that the neonatal challenge was designed to directly evaluate protective efficacy following maternal mRNA-LNP administration. To make this explicit, we revised the Methods (**line 208 - 218**) to clearly state that intestinal bacterial counts were measured as an indicator of protection in pups following challenge. We have also updated our results to clarify that levels of ZAC-3 IgG1 and IgA were not sufficient to limit intestinal colonization of *V. cholerae* O1 when pups were separated from dams following intragastric challenge; however, a significant reduction in bacterial burden was observed when challenged pups remained with dams for continuous breastfeeding.

**1.3 The use of dams and pups is appropriate, but more detail on animal numbers, age, and selection criteria would strengthen reproducibility. Moreover, Sample sizes are modest; statistical power is not discussed, making it unclear whether negative findings (e.g., lack of intestinal protection) reflect biology or underpowered experiments.** Response: We have now included information on animal numbers/sample size to the Methods section, as well as the Figure legends. The inherent challenges of working with newborn mice limits sample sizes, but we have overcome those challenges with multiple dams and litters.

Therefore, our sample sizes in this study are indeed modest and we have included data to show that returning pups to dams for continuous feeding after challenge significantly reduces bacterial burden in gut tissues **(Fig. 6)**. Furthermore, we have expanded the Statistical Analysis section **(line 223 -230)** to clarify the assumptions tested and the adjustment for multiple comparisons. Statistical analysis was applied only to Figure 2B and Fig 6, which involved a direct comparison between two independent biological groups (lactating vs non-lactating mice for Fig 2B; and IgG/IgA vs Naïve). All other figures did not undergo statistical testing because several assays utilized pooled samples (e.g., vibriocidal assays and litter-level pooled samples), which are not independent biological replicates and therefore not suitable for hypothesis testing. In these cases, results are presented descriptively, as with results showing the differences between naïve and experimental conditions where naïve groups had undetectable activity and where the differences were evident from the graphed data.

**1.4: Ethical approval is mentioned, but more detail on animal welfare monitoring and adverse effect assessment would provide reassurance.** Response: We have added the following text: The animals were monitored daily for any adverse events by trained research personnel. Similarly, dams were observed for 30 min after each immunization for potential adverse reactions. After anesthesia and milk collection, dams were monitored continuously until full recovery and normal behavior were confirmed. Dam–pup reunions were supervised for 30 min to ensure appropriate maternal behavior and absence of pup rejection. Pups undergoing challenge were assessed every 2 h between during normal business hours to confirm tolerance of the procedure.

**RESULTS**

Reviewer #1:

**1.1** **Broadly yes, but the study leaves key mechanistic questions unresolved (e.g., FcRn role, SIgA formation, antibody persistence). Additional experiments would better align results with the stated goals**. Response: We completely agree that the investigation of certain mechanistic pathways – including FcRn-mediated transport, SIgA formation, and the basis for antibody persistence – would greatly enhance the study. However, the neonatal model of *V. cholerae* we employed comes with notable technical challenges and beyond our capacity with existing resources.

Notwithstanding, address the relevant point, we revised the Discussion section to ensure that our statements remain descriptive and do not imply mechanistic conclusions beyond the data collected. Where relevant we now explicitly state that these aspects remain to be investigated in future work.


**1.2 The main findings are clearly shown, but presentation is incomplete. Absolute antibody concentrations are missing, protective efficacy is not directly demonstrated, and mechanistic claims sometimes exceed the data.** Response: To address the concern regarding missing absolute antibody concentrations, we revised the relevant figures and figure legends to include concentrations in µg/mL wherever applicable. Absolute concentrations (µg/mL) are shown in the corresponding isotype-specific ELISA data, which represent the same samples used in the whole-cell ELISAs. Because whole-cell ELISAs inherently yield OD values rather than absolute concentrations, we now clarify this relationship in the figure legends for transparency.

Regarding protective efficacy, we clarify in the Methods and Results that the neonatal challenge assay was designed to directly evaluate protection. We now include data demonstrating that continuous breastfeeding significantly reduces intestinal bacterial burden in pups nursed by dams that received either IgG or IgA mRNA (Fig. 6), and we discuss these findings accordingly in the Discussion.

Finally, as addressed in our response to the preceding comment, we revised the Discussion to ensure that statements regarding mechanistic pathways remain descriptive and do not exceed the data collected.

**1.3** **Figures are generally clear but should include absolute concentrations (µg/mL) and more precise labeling to enhance reproducibility and interpretation.** Response: As noted above, we revised all relevant figure panels and legends to include absolute antibody concentrations (µg/mL) where applicable, and we clarified the relationship between OD-based whole-cell ELISAs and the corresponding isotype-specific ELISA concentrations. We also refined figure labels and legend descriptions to improve clarity and reproducibility.

**Conclusions**

Reviewer #1:

**1.1 Partially. The data convincingly demonstrate maternal-to-infant transfer of mRNA-encoded IgG; however, claims of protective efficacy are overstated, given the lack of intestinal protection or challenge studies.** Response: We agree that the data support robust maternal-to-infant transfer of mRNA-encoded ZAC-3 IgG. We have now included data demonstrating that further optimization; specifically, continuous breastfeeding reduces intestinal bacterial burden in pups nursed by dams that received either IgG or IgA mRNA (Fig. 6), and we discuss these findings accordingly in the Discussion.

**1.2 Are the limitations of the analysis clearly described**? **Some are acknowledged (e.g., uncertainty about antibody persistence), but key gaps—such as the failure to generate SIgA, the absence of protective benchmarks, and limited mechanistic insight—require a fuller discussion.** Response: As reflected in the revised text (Lines 611 - 618), we now explicitly clarify that SIgA formation was not assessed in this study, that IgA2 expression was too low to characterize polymeric forms, and that mechanistic pathways such as FcRn-mediated transport remain speculative. We have now included data demonstrating that further optimization; specifically, continuous breastfeeding reduces intestinal bacterial burden in pups nursed by dams that received either IgG or IgA mRNA (Fig. 6), and we discuss these findings accordingly in the Discussion. These revisions ensure that our interpretations remain descriptive and appropriately constrained by the data.

**1.3. Do the authors discuss how these data can contribute to advancing our understanding of the topic under study? Yes. They highlight the novelty of mRNA-LNP–driven antibody transfer via milk; however, the discussion could be improved by better connecting mechanistic insights (e.g., trafficking, FcRn role) to the translational potential.** Response: In the revised Discussion, we added clarifying statements to more explicitly connect our observations – particularly regarding antibody distribution, trafficking to milk, and potential FcRn involvement – to the broader translational implications of mRNA-encoded antibody platforms. These additions are purely contextual and do not make mechanistic claims beyond the data. They emphasize how understanding maternal antibody trafficking pathways will be important for optimizing future mRNA-based passive immunization strategies. These revisions appear in lines 408-424.

**Is public health relevance addressed?**

**1.4. Yes, the potential for infant protection against emerging pathogens is emphasized, but claims should be tempered until efficacy is demonstrated in relevant challenge models.** Response: We acknowledge the reviewer’s point that, although the public health relevance of maternal mRNA-delivered antibodies is high, any implications for infant protection must be framed cautiously until efficacy is demonstrated in appropriate challenge models. In response, we revised the Abstract, Introduction, and Discussion to ensure that statements about potential impact are appropriately qualified and do not imply protective efficacy beyond what was observed experimentally. These clarifications appear in **lines 31–35, 87–92, and 437–441**. The revised text emphasizes that while this platform shows promise for enhancing maternal and infant immunity, protective efficacy in vivo was achieved in this study when the pups were allowed to continue breastfeeding and will require further optimization and validation in future pathogen-specific challenge models.

Reviewer #2: see below

Reviewer #3: (No Response)

**Editorial and Data Presentation Modifications?**

**Summary and General Comments**

Reviewer #1:

**1.1 The manuscript explores whether maternal delivery of mRNA-LNPs encoding mAbs can generate protective titers in breast milk and transfer immunity to offspring. Using the anti-V. cholerae antibody ZAC-3, the authors demonstrate the systemic and milk expression of mRNA-encoded IgG and the maternal-to-infant transfer of this antibody. To date, this is the first direct evidence of mRNA-encoded antibodies passing from mother to pups. The study is well-written, conceptually novel, and relevant, particularly for infant protection against emerging pathogens. However, important clarifications, additional data, and more nuanced interpretations are needed. While it establishes proof of concept, it does not demonstrate protective efficacy against enteric challenge, limiting translational claims. The lack of intestinal protection highlights the need for optimized antibody formats (e.g., SIgA) and a deeper understanding of antibody trafficking and persistence. The manuscript is suitable for publication after major revision, with emphasis on tempering claims of efficacy, clarifying IgA biology, and strengthening data on antibody localization and function.**

Response: We thank the reviewer for their thoughtful evaluation of our manuscript and for recognizing the novelty and relevance of demonstrating maternal-to-infant transfer of an mRNA-encoded monoclonal antibody. We also appreciate the concerns raised regarding mechanistic interpretation, IgA biology, and the need to temper statements of protective efficacy.

In response, we have made substantial revisions throughout the Abstract, Introduction, Results, Discussion, and figure legends. These revisions (1) include data demonstrating that further optimization; specifically- continuous breastfeeding - reduces intestinal bacterial burden in pups nursed by dams that received either IgG or IgA mRNA (Fig. 6), and we discuss these findings accordingly in the Discussion;and (2) ensure that mechanistic explanations regarding antibody trafficking, FcRn function, and IgA biology are presented cautiously and without overinterpretation. We believe these changes address the reviewer’s concerns and improve the accuracy, transparency, and translational framing of the manuscript. Detailed responses to each comment, along with corresponding line-by-line revisions, are provided below.

Major concerns
**1.2 The study shows detectable ZAC-3 IgG in maternal milk and pup serum, but not in intestinal homogenates. This observation is critical: protection against *V. cholerae* requires mucosal antibodies, preferably secretory IgA. The hypothesis that FcRn-mediated transport sequesters IgG into the systemic compartment at the expense of luminal protection is plausible, but requires direct evidence (e.g., antibody quantification in intestinal contents at different ages, FcRn knockout controls, or blocking FcRn function). Without such data, the mechanistic conclusion remains speculative.** Response:

We agree that protection against *V. cholerae* requires sufficient antibody levels in the intestinal lumen and that secretory IgA is the biologically optimal isotype for enteric defense. We also recognize that the absence of detectable ZAC-3 in intestinal homogenates of either dams or pups limits direct conclusions regarding steady-state mucosal antibody levels. Importantly, our study evaluated both ZAC-3 IgG1 and IgA2 mRNA constructs in parallel. While the IgG1 construct yielded robust expression in serum and breast milk of dams, IgA2 expression was approximately ten-fold lower and was not detectable in intestinal tissues despite targeted screening. With the inclusion of new intragastric challenge data, our findings further suggest that intestinal antibody availability in this model is highly context dependent. In the absence of luminal antigen, antibodies delivered through milk may be rapidly absorbed into circulation, consistent with the detection of IgG in pup serum. In contrast, when pups were continuously breastfed during active *V. cholerae* challenge, we observed a significant reduction in bacterial burden compared to naive, indicating that antibody-target interaction in the intestinal lumen may promote functional retention and protection. We therefore interpret the absence of detectable gut antibody under non-challenge conditions not as evidence of failed delivery, but rather as a reflection of rapid redistribution in the absence of antigen engagement. We have incorporated this interpretation into the Discussion to ensure that our conclusions regarding mucosal protection remain biologically grounded and aligned with the updated experimental findings. Regarding FcRn-mediated transport, we agree that the proposed mechanism remains speculative without direct experimental evidence. The intent of this discussion was to offer a plausible biological explanation for the absence of luminal IgG, not to assert a mechanistic conclusion. In response, we have revised the Discussion to (1) explicitly frame FcRn involvement as a hypothesis, (2) emphasize that neither FcRn function nor intestinal permeability was directly measured, and (3) clarify that confirming these pathways would require additional experiments, such as luminal antibody quantification across developmental time points or evaluation in FcRn-deficient models. We have incorporated these clarifications into the Discussion **(lines 31-35, 87-92, 140-150, 210-215, 356-360, 426-439)**, ensuring that interpretations remain aligned with the experimental data.

**1.3 : The finding of much lower expression of IgA2 versus IgG1 highlights a major challenge. However, the study does not address whether dimeric/secretory IgA was successfully formed, or whether constructs included J-chain or supported polymerization. Given the known superiority of SIgA in mucosal defense, demonstrating the feasibility of mRNA-mediated SIgA production (rather than monomeric IgA) is essential for the translational relevance of this platform to enteric infections.** Response: We thank the reviewer for raising this point and agree that secretory IgA is a critical mediator of mucosal defense against enteric pathogens. The use of mRNA to successfully encode SIgA was addressed in a previous publication [Deal et al. 10.1016/j.xcrm.2023.101253]. In the current study, the IgA2 mRNA-LNP formulation was designed exactly the same way to support dimeric IgA assembly and included mRNA encoding the ZAC-3 IgA2 heavy chain, light chain, and joining chain (JC) at a 4:4:1 ratio. However, we did not directly assess J-chain incorporation, polymerization state, or formation of secretory IgA. Our analyses were limited to measuring total human IgA levels in serum and breast milk and determining whether IgA could be detected in intestinal tissues. We have clarified these points in the Discussion.

**1.4 The authors note uncertainty about whether sustained antibody detection reflects long-lived IgG versus ongoing translation from residual mRNA. This is an important mechanistic point and warrants direct investigation. Suggested approaches include: (a) Tracking mRNA degradation by RT-qPCR in tissues. (b) Measuring antibody decay rates after single vs. repeated administrations. (c) Without such data, the persistence claims remain incomplete.** Response: We appreciate the reviewer’s insight regarding the distinction between persistence of circulating antibody and the underlying mechanisms responsible for that persistence. As noted in the Discussion, our study was not designed to differentiate whether sustained detection of ZAC-3 IgG reflects ongoing translation of residual mRNA or the inherent serum half-life of IgG1. We agree that resolving this question is important for understanding the kinetics and durability of mRNA-encoded antibodies.

At present, we did not perform experiments to quantify mRNA decay in tissues or to measure antibody clearance rates following single versus repeated administrations. Because our focus in this proof-of-concept study was to evaluate antibody expression, milk transfer, and neonatal exposure after a single maternal dose, mechanistic dissection of mRNA or antibody kinetics was beyond the intended scope. To address this point, we have revised the Discussion to clearly acknowledge that the basis for antibody persistence remains unresolved in this model, and that future studies will be required to (a) directly measure mRNA degradation kinetics in relevant tissues and (b) define IgG clearance rates following mRNA administration. These studies will be essential for establishing dose–response relationships and optimizing the durability of mRNA-based maternal immunization approaches.

**1.5 The study does not establish the quantitative thresholds of mucosal antibody required to block colonization. Passive transfer experiments with purified ZAC-3 IgG/IgA at defined doses (administered by oral gavage to pups) would help establish protective levels.** Response: Although establishing protective dose–response relationships was not an objective of the present maternal mRNA–LNP study, this topic has been partly assessed in prior work from our laboratory. First, previous passive-transfer studies performed in our laboratory demonstrated that premixing purified ZAC-3 IgG with *V. cholerae* and delivering it orally to neonatal mice results in dose-dependent reductions in intestinal colonization, defining the approximate luminal antibody concentrations required for protection (unpublished data). Second, Baranova et al. (2020; DOI:10.20411/pai.v5i1.370) similarly established that milk-derived ZAC-3 IgA1 at defined concentrations (20–40 µg/mL in stomach/intestine) provides significant protection against cholera challenge in newborn mice (2-log CFU reduction) (**Figures 3,4**). These findings further support the concept that ZAC-3–mediated protection depends on achieving sufficient luminal antibody concentrations. In the present study, ZAC-3 IgG1 and IgA delivered via maternal mRNA–LNP administration was not detectable in intestinal homogenates of dams or pups, indicating that local concentrations were below previously defined protective thresholds in the absence of continuous breastfeeding; but concentration builds up as feeding continues (as explained earlier in the response above). We have added clarifying language in the Discussion.

**Minor concerns**

**1.6: The manuscript should clearly distinguish between the detection of antibodies in serum/milk and protective efficacy in vivo. Currently, the narrative risks overinterpreting findings as proof of protection when it was not achieved**. Response: The manuscript now clearly distinguishes between antibody detection, maternal transfer, and in vivo protective efficacy. We explicitly state that antibody expression and transfer alone were not sufficient to confer protection in the initial neonatal challenge model in which pups were separated from dams. Importantly, with the inclusion of the continuous breastfeeding challenge model, we now show that sustained exposure to ZAC-3 IgG via milk was associated with a significant reduction in intestinal V. cholerae burden, whereas IgA produced only a modest, non-significant effect. The text has been revised throughout to ensure that protection is discussed only in the context in which it was experimentally observed, and that antibody detection alone is not presented as evidence of efficacy. These revisions prevent overinterpretation and more accurately reflect the conditional nature of protection in this model.
**1.7** **The discussion of neonatal gut permeability vs. FcRn transport is appropriate but should be framed as a hypothesis rather than a firm conclusion**. Response: We have revised the Discussion to ensure that the descriptions of neonatal gut permeability and FcRn-mediated transport are presented as hypotheses rather than conclusions. These mechanisms are now explicitly framed as speculative and not directly tested in this study.

**1.7 It would be valuable to clarify whether any adverse effects were observed in dams or pups following mRNA-LNP administration (e.g., inflammation, growth delay).** Response: No adverse effects were observed in either dams or pups following mRNA-LNP administration. Throughout the study, animals showed normal activity, nursing behavior, body condition, and survival, and no abnormalities were noted during routine monitoring. We have added a brief statement to the Methods (Animal Procedures) to clarify that no overt physiological or behavioral changes were detected.

**1.8 Figures and tables should include absolute concentrations (µg/mL), not just relative values, to facilitate reproducibility and inter-study comparison.** Response: We confirm that absolute antibody concentrations (µg/mL) have now been added to the figures and legends wherever applicable to ensure reproducibility and facilitate comparison across studies.

**Reviewer #2: Very interesting**
To my knowledge, first use of mRNA technology to try to induce antigen-specific Ab in breast milk to protect newborns (in this case pups) Long known that vaccinating moms can boost breast milk Ig, both IgG and in primed moms/previously infected, IgA. In this study, the attempt was to induce high level responses in breast milk in not primed used a cholera model Pros: non invasive bacterial pathogen, breast milk protects, there was a well characterized mAb Cons: cholera doesn’t really affect under 18-24 months (in part because in cholera endemic areas, breast milk is the source of hydration, not contaminated water But for the purpose of a model system, perfectly reasonable The researchers achieved significant IgG levels in moms/dams, less so IgA. IgG was in breast milk. Made it into pups The IgG had vibriocidal activity (suggesting correct confirmation) in the model system, did not change the CFU of intestinal V cholerae in challenged pups

A few thoughts: the manuscript would be strengthened by discussing/addressing/clarifying below

**2.1 Why give IV, why not IM for more prolonged expression.** Response: Intravenous (IV) administration was selected for this proof-of-concept study because it yields rapid and high systemic expression of secreted mRNA-encoded antibodies. This allowed us to tightly control the timing of ZAC-3 expression relative to milk collection, pup exposure, and neonatal challenge, which was essential for assessing maternal expression and transfer dynamics.

We agree that intramuscular (IM) delivery can result in prolonged expression and may influence antibody kinetics differently. While IM administration could also be explored in this model, our goal in the present study was to establish whether maternal mRNA-LNP delivery can generate functional antibodies that appear in milk and can be transferred to offspring independent of administration route within the study window. We have added a statement to the Methods explaining the rationale for the selected route of administration.

**2.2 IV not practical in emergency situation.** Response: We agree that intravenous administration may not always be practical in emergency or large-scale deployment scenarios. In this study, IV delivery was used as a controlled, preclinical approach to establish proof of concept for maternal expression and transfer of mRNA-encoded antibodies within a defined timeframe. The choice of IV administration was intended to ensure rapid and reproducible systemic antibody expression rather than to propose a clinically optimized delivery strategy. We have clarified in the manuscript that route of administration was selected to address the specific experimental objectives of this study.

**2.3 What cell type is making antibody? Most Ig in breast milk is produced locally in breast tissue, why would you think IV would give breast milk Ig.** Response: We agree that the majority of naturally derived antibodies in breast milk – particularly IgA – are produced locally by plasma cells resident in mammary tissue. However, our study does not propose local antibody production within the mammary gland. Following intravenous administration, mRNA–lipid nanoparticles are known to preferentially drive protein expression in the liver and other highly perfused tissues, resulting in systemic production of the encoded antibody (Pardi et al., 2015). We therefore interpret the presence of ZAC-3 IgG in breast milk in our studies as reflecting transfer from the systemic circulation rather than local synthesis within mammary tissue. IgG can be transferred from the systemic circulation into breast milk via FcRn-mediated transport across mammary epithelial cells, a mechanism that has been well described in rodents (Roopenian and Akilesh, 2007). This pathway provides a means by which circulating IgG can appear in milk independent of local antibody production.

**2.4 Any evidence your IgA actually made with JC, and any staining of breast tissue for production?** Response: As noted above in response to Reviewer 1, The use of mRNA to successfully encode SIgA was addressed in a previous publication [Deal et al. 10.1016/j.xcrm.2023.101253]. In the current study, the IgA2 mRNA-LNP formulation was designed exactly the same way to support dimeric IgA assembly and included mRNA encoding the ZAC-3 IgA2 heavy chain, light chain, and joining chain (JC) at a 4:4:1 ratio. However, we did not directly assess J-chain incorporation, polymerization state, or formation of secretory IgA. Our analyses were limited to quantifying total human IgA in serum and breast milk and determining whether IgA could be detected in intestinal tissues and provide protection.

We did not perform staining of mammary tissue for local antibody production, as the study was not designed to investigate mammary gland synthesis of ZAC-3 antibodies. Instead, the focus was on systemic expression following mRNA-LNP delivery and the subsequent appearance of antibodies in serum and milk. We have clarified these points in the Discussion to avoid any implication that J-chain–containing dimeric or secretory IgA was directly demonstrated in this model.

**2.5 Define HC, LC, JC for reader.** Response: As requested, we have defined HC, LC, and JC at their first occurrence in the Methods section.

**2.6 My memory is rodents pump dIgA from blood into biliary system; does same happen with breast?** Response: Correct, in rodents, dimeric IgA produced systemically can be transported to the biliary system via pIgR-mediated transcytosis in hepatocytes and delivered into intestinal secretions. Dimeric IgA is also transported across mammary gland epithelium although the efficacy of human IgA2 transport relative to mouse dIgA is unknown and may account for reduced levels in milk, especially considering that De Groot (2000) argued that pIgR is rate limiting in mammary transport [10.1046/j.1365-2567.2000.00094.x]. We have not mentioned this point in the text.

**2.7 Why did you give a single VLP [LNP] with both an IgG and an dIgA mRna. Why not do separately?** Response: We thank the reviewer for this question and would like to clarify that IgG1 and IgA2 mRNA were not packaged together in a single LNP. Instead, ZAC-3 IgG1 and ZAC-3 IgA2 were encoded by separate mRNA constructs and formulated into distinct mRNA-LNP preparations, which were administered and evaluated independently.

**2.8: ELISA protocol, seems like decribed IgG capture/detection, please add the IgA methods.** Response: The isotype-specific ELISA methods used to quantitate both human IgG and IgA in serum and breast milk were described together in the Methods section.

**2.9: line 379 missing ‘antibody.** Response: We thank the reviewer for catching this typographical omission. We have corrected the text.

**2.10: Why do WC Elisa and not LPS.** Response: Whole-cell ELISA was chosen as an established and widely used assay for assessing antibody binding to *V. cholerae* surface antigens in their native context, including LPS presented in the intact outer membrane. Both whole-cell and LPS-based ELISAs are accepted approaches for evaluating anti-*V. cholerae* antibodies, particularly those targeting LPS epitopes. Because ZAC-3 recognizes the conserved core region of *V. cholerae* LPS, and because functional activity was independently confirmed using complement-dependent vibriocidal assays, whole-cell ELISA provided an appropriate and biologically relevant binding readout for this study.

**2.11 Judging protection used presence of V cholerae in intestine, but no evidence anti-LPS antibodies kill the bacteria so the assay may not be the optimal one.** Response: The mRNA construct used in this study encodes the well-characterized anti–*V. cholerae* core LPS monoclonal antibody ZAC-3; therefore, any antibody expressed from this mRNA is, by definition, LPS-specific. This is consistent with the observed binding to intact *V. cholerae* in whole-cell ELISAs and with prior work defining the antigen specificity of ZAC-3. Importantly, protection against *V. cholerae* in the intestinal lumen does not require direct bacterial killing by antibodies. Anti-LPS antibodies such as ZAC-3 have been shown to mediate protection primarily by inhibiting intestinal colonization through mechanisms including agglutination and interference with bacterial attachment, rather than complement-dependent lysis, which is limited in the gut environment. Moreover, prior work has demonstrated that ZAC-3 reduces *V. cholerae* intestinal colonization in vivo when present at sufficient levels, using both IgG (unpublished results) and IgA versions of ZAC-3 (Baranova et al. (2020; DOI:10.20411/pai.v5i1.370). For this reason, quantification of intestinal bacterial burden is a standard and appropriate endpoint for assessing protection in neonatal cholera challenge models.

In the present study, although mRNA-encoded ZAC-3 IgG was biologically functional in vitro, antibody levels in the intestinal compartment of pups were not detectable under the initial challenge conditions in which pups were separated from dams, and no reduction in intestinal colonization was observed. However, when pups were returned to dams for continuous breastfeeding following challenge, a significant reduction in intestinal *V. cholerae* burden was observed in the IgG group and modestly so for the IgA group (consistent with the substantially lower IgA levels achieved in milk and pups), demonstrating that protection can be achieved when sustained milk-derived antibody delivery is maintained in the presence of antigen. We therefore interpret the initial lack of protection as reflecting insufficient luminal antibody availability under limited feeding conditions, rather than a limitation of the protection assay itself or of ZAC-3 antibody function.

**2.12: Fig 2 demonstrates a profound effect of lactation status, would discuss more in discussion. the milking mice protocol was a tour de force**. Response: We thank the reviewer for this observation. We have expanded the discussion to more carefully contextualize the effect of lactation status on circulating ZAC-3 IgG levels. Specifically, we describe the lower serum concentrations observed in lactating dams relative to non-lactating mice and discuss how physiological changes associated with pregnancy and lactation may influence antibody biodistribution and pharmacokinetics. Importantly, we frame these findings descriptively and avoid assigning a specific mechanistic explanation, noting that the study was not designed to determine whether differences reflect redistribution, altered clearance, or accumulation in specific tissues.

**2.13: Figures only show IgG, please add IgA.** Response: All figures now show IgA graphs along with IgG except Figure 4 because IgA could did not reach detectable levels in pup serum.

**Reviewer #3:**

This is a well-written and thoughtfully designed study that explores the feasibility of using mRNA-LNP technology to express and deliver pathogen-specific monoclonal antibodies (mAbs) through breast milk to newborns. The authors use a Vibrio cholerae model and the well-characterized ZAC-3 monoclonal antibody, providing clear experimental evidence of successful expression in maternal serum and milk, and transfer to pups. The manuscript addresses an important gap in maternal-infant immunology and proposes an innovative platform for passive immunization in outbreak or vaccine-limited settings. The approach is conceptually strong, and the experimental framework is well executed. However, some aspects of experimental design, data interpretation, and mechanistic depth could be strengthened to increase the impact and clarity of the work.

Major Weaknesses and Recommendations
**3.1. Although functional antibodies were detected in maternal serum, milk, and pups, no reduction in V. cholerae colonization was observed. This discrepancy warrants further investigation. The authors could consider using a higher dose, repeated mRNA administrations, or an alternative route of administration to achieve protective antibody levels.** Response: We thank the reviewer for this comment. In the revised manuscript, we now clarify that the initial lack of protection was observed under conditions in which pups were separated from dams following challenge, limiting sustained luminal antibody exposure. Importantly, when pups were returned to dams for continuous breastfeeding after infection (Fig. 6), a significant reduction in intestinal V. cholerae burden was observed in the IgG group. We have revised the Discussion to reflect this distinction and to contextualize protection as dependent on sustained antibody delivery and luminal availability rather than antibody functionality alone. These revisions clarify the relationship between antibody concentration, exposure dynamics, and protective efficacy in this model without making mechanistic claims beyond the data.

**3.2. The authors hypothesize FcRn-mediated transfer but do not directly evaluate it. Including supporting experiments (e.g., FcRn blockade, knockout models, or tracking antibody localization) or providing a more detailed discussion of how FcRn-mediated transport might limit gut availability would strengthen the manuscript.** Response: We agree that FcRn-mediated transport was not directly evaluated in this study and that experimental interrogation of this pathway would require dedicated models beyond the scope of the current work. In response, we have revised the Discussion to ensure that FcRn involvement is presented strictly as a hypothesis and not as a mechanistic conclusion. We now explicitly state that FcRn-mediated transport was not tested experimentally and that the absence of detectable antibody in intestinal homogenates is an observational finding without direct mechanistic attribution **(lines 440 - 456)**. These revisions ensure that interpretations remain descriptive and appropriately bounded by the data generated in this study.

**3.3. IgA expression was low and only briefly analyzed. Given the mucosal protective role of IgA, the authors should provide more quantitative details and consider discussing potential approaches to optimize IgA expression or delivery.** Response: Quantitative IgA data are now provided in the Results and these findings are clearly described in the main text. In response to the reviewer’s suggestion, we have expanded the Discussion to explicitly address potential factors that may influence IgA expression and delivery following mRNA-based administration. Specifically, we note that achieving higher mucosal IgA levels may require optimization of dosing strategies and route of administration to influence expression magnitude, duration, and tissue distribution **(lines 484–490)**.

**3.4. Some figures rely on pooled samples or have small n, which limits statistical power. The authors should provide exact n values for each experiment in the figure legends and display individual data points to enhance transparency and statistical robustness.** – We have now included samples sizes in Methods section, Results and Figure legends as appropriate.

**3.5. The study is innovative and relevant for outbreak response; however, the translational implications are underdeveloped. Expanding the discussion on potential clinical applications, safety considerations, and how this platform could integrate with existing maternal vaccination strategies would strengthen the manuscript’s impact.**

Response: We thank the reviewer for highlighting the importance of strengthening the translational perspective. In the revised Discussion (lines 558 - 571), we have expanded the clinical framing of this platform by emphasizing its potential application for systemic neonatal infections such as sepsis, pneumonia, and meningitis, particularly in settings where maternal immunization occurs after delivery. We also discuss key translational considerations, including antibody persistence, mRNA stability, and the need to distinguish between continued mRNA translation and intrinsic antibody half-life. These additions clarify how this approach could complement existing maternal vaccination strategies by providing a rapid, postnatal passive immunization option during outbreaks, while also acknowledging current safety and durability knowledge gaps that must be addressed for clinical translation.

**3.6 Also, the current title “Delivery of a protective Vibrio cholerae-specific, mRNA-derived monoclonal IgG into mouse breast milk” is potentially misleading, as the study does not demonstrate actual protection against V. cholerae challenge in neonatal mice. For accuracy and transparency, it would be more appropriate to revise the title to reflect the demonstrated findings rather than the intended goal.**

Response: We have revised the title to more accurately reflect the demonstrated findings of the study. The title now reads: “Maternal expression and breast milk transfer of an mRNA- monoclonal antibody in a murine model of cholera”.

**3.6. While antibody expression and transfer were clearly demonstrated, the lack of mucosal protection highlights critical challenges in achieving protective antibody titers at the mucosal surface. Addressing these mechanistic gaps, particularly regarding FcRn-mediated transport and mucosal localization, would significantly strengthen the manuscript and enhance its translational impact. The study is conceptually strong and technically solid but would benefit from additional analysis or expanded discussion to explain the lack of protective effect and to better define the translational significance of the findings.** Response: In the revised Discussion, we included data showing that continuous breastfeeding following challenge allows for protective activity, supporting the concept that luminal antibody exposure in the presence of antigen could be a key determinant of functional efficacy. Together, this addition refines the mechanistic interpretation of our results and better define the translational implications of the platform.

In terms of more detailed follow-up experiments, we are, at least for the time being, resource constrained and unable to conduct further investigation into detailed mechanisms of transport. While we do not explicitly state that in the text, we note from the Abstract onwards that “ Our findings highlight the potential of mRNA-based monoclonal antibody platforms in the maternal-newborn context, while acknowledging the need for optimized antibody isotypes, dosing, and tissue-specific delivery to improve mucosal immunity.” It is our sincere hope that more detailed studies will be undertaken in the near future.

**Figure resubmission:** While revising your submission, we strongly recommend that you use PLOS’s NAAS tool (<https://ngplosjournals.pagemajik.ai/artanalysis>) to test your figure files. NAAS can convert your figure files to the TIFF file type and meet basic requirements (such as print size, resolution), or provide you with a report on issues that do not meet our requirements and that NAAS cannot fix.

  After uploading your figures to PLOS’s NAAS tool - <https://ngplosjournals.pagemajik.ai/artanalysis>, NAAS will process the files provided and display the results in the "Uploaded Files" section of the page as the processing is complete. If the uploaded figures meet our requirements (or NAAS is able to fix the files to meet our requirements), the figure will be marked as "fixed" above. If NAAS is unable to fix the files, a red "failed" label will appear above. When NAAS has confirmed that the figure files meet our requirements, please download the file via the download option, and include these NAAS processed figure files when submitting your revised manuscript.

**Reproducibility:** To enhance the reproducibility of your results, we recommend that authors of applicable studies deposit laboratory protocols in protocols.io, where a protocol can be assigned its own identifier (DOI) such that it can be cited independently in the future. Additionally, PLOS ONE offers an option to publish peer-reviewed clinical study protocols. Read more information on sharing protocols at <https://plos.org/protocols?utm_medium=editorial-email&utm_source=authorletters&utm_campaign=protocols>
